# Catalytic Conversion of Lipophilic Substrates by Phase constrained Enzymes in the Aqueous or in the Membrane Phase

**DOI:** 10.1038/srep38316

**Published:** 2016-12-05

**Authors:** Marcus Cebula, Ilke Simsek Turan, Birgitta Sjödin, Madhuranayaki Thulasingam, Joseph Brock, Volodymyr Chmyrov, Jerker Widengren, Hiroshi Abe, Bengt Mannervik, Jesper Z. Haeggström, Agnes Rinaldo-Matthis, Engin U. Akkaya, Ralf Morgenstern

**Affiliations:** 1 Institute of Environmental Medicine, Karolinska Institutet, Nobels väg 13, 17177, Stockholm, Sweden; 2 UNAM-Institute of Material Science and Nanotechnology, Bilkent University, Bilkent, Ankara, 06800, Turkey; 3 Department of Neurochemistry, Stockholm University, Svante Arrhenius väg 16C, 10691, Stockholm, Sweden; 4 Department of Medical Biochemistry and Biophysics, Karolinska Institutet, Scheeles väg 2, 17177, Stockholm, Sweden; 5 Experimental Biomolecular Physics, Royal Institute of Technology-KTH, Albanova, Roslagsvägen 30 B, 11419, Stockholm, Sweden; 6 Department of Chemistry, Graduate School of Science, Nagoya University, Furo-cho, Chikusa-ku, Nagoya 464-8602, Japan; 7 Department of Chemistry & UNAM-Institute of Material Science and Nanotechnology, Bilkent University, Bilkent, Ankara, 06800, Turkey

## Abstract

Both soluble and membrane-bound enzymes can catalyze the conversion of lipophilic substrates. The precise substrate access path, with regard to phase, has however, until now relied on conjecture from enzyme structural data only (certainly giving credible and valuable hypotheses). Alternative methods have been missing. To obtain the first experimental evidence directly determining the access paths (of lipophilic substrates) to phase constrained enzymes we here describe the application of a BODIPY-derived substrate (PS1). Using this tool, which is not accessible to cytosolic enzymes in the presence of detergent and, by contrast, not accessible to membrane embedded enzymes in the absence of detergent, we demonstrate that cytosolic and microsomal glutathione transferases (GSTs), both catalyzing the activation of PS1, do so only within their respective phases. This approach can serve as a guideline to experimentally validate substrate access paths, a fundamental property of phase restricted enzymes. Examples of other enzyme classes with members in both phases are xenobiotic-metabolizing sulphotransferases/UDP-glucuronosyl transferases or epoxide hydrolases. Since specific GSTs have been suggested to contribute to tumor drug resistance, PS1 can also be utilized as a tool to discriminate between phase constrained members of these enzymes by analyzing samples in the absence and presence of Triton X-100.

The subcellular localization of enzymes underlies the compartmentalization of metabolic processes. In all cellular compartments soluble and membrane-bound enzymes coexist and a rationale for an aqueous or lipid localization is often taken for granted. Simply put, soluble enzymes tend to use hydrophilic substrates and membrane-bound enzymes lipophilic ones. There are however, classes of enzymes acting on lipophilic substrates that have members in both phases such as the xenobiotic-metabolizing sulphotransferases/UDP-glucuronosyl transferases, epoxide hydrolases or glutathione transferases^1^,^2^,^3^,^4^. The dual location ensures efficient removal of toxic and reactive xenobiotics. What then are the distinguishing mechanistic features of enzymes from the two phases? A set of general mechanistic alternatives have been outlined based on structural data (for a wide selection of enzymes) including intramembrane or external substrate access for membrane proteins or intramembrane access for soluble enzymes^5^. However, as the authors point out, experimental evidence for the proposed access paths is still lacking. To address these issues, we here studied glutathione transferases (GSTs) and their interaction with lipophilic substrates. GSTs are major phase II metabolizing enzymes that predominantly catalyze the conjugation of reduced GSH to a wide range of hydrophobic, endogenous and exogenous, electrophilic molecules. The GSTs are divided into phylogenetically distinct soluble and membrane bound microsomal families that each contain many isoforms. Importantly, the soluble and membrane bound enzymes display specific as well as overlapping substrate specificities^1^,^6^,^7^. Using substrates with varying degrees of lipophilicity that cover a broad range of logP including one substrate that can uniquely probe the aqueous or membrane phase accessibility, we experimentally demonstrate that cytosolic and membrane-bound microsomal GSTs have a limited capacity to reach hydrophobic substrates in their opposing phases. Furthermore we suggest that membrane embedded enzymes benefit from the pronounced enrichment of lipophilic substrates at the phospholipid headgroup/hydrocarbon chain intersection - a suggestion that is supported by structural data^8^.

## Results and Discussion

### Conversion of lipophilic substrates by cytosolic GSTs

The statement that cytosolic enzymes act most efficiently on substrates in the aqueous phase might seem obvious. However, for cytosolic enzymes acting on hydrophobic substrates this needs to be experimentally verified. We therefore studied how cytosolic GSTs catalyze the conjugation of substrates with varying degrees of hydrophobicity using detergent as a membrane mimic. In a two phase system (water/detergent) the substrate concentration will rapidly reach equilibrium between the hydrophilic and hydrophobic phases dependent on its lipophilicity (characterized by the partition coefficient logP). As a consequence, the turnover of a cytosolic enzyme will be reduced if it only has access to the substrate (concentration) in the aqueous phase (the aqueous concentration being low compared to the one in detergent mimicking the *in vivo* situation). While enzyme turnover is consequently reduced in the presence of detergent cytosolic enzymes still have the capacity to conjugate all molecules as the equilibrium will continuously replenish molecules from the hydrophobic phase to the hydrophilic phase. Of course, this behavior only holds true for molecules that are able to move between phases, i.e. that do not partition close to 100%.

**Figure 1:**
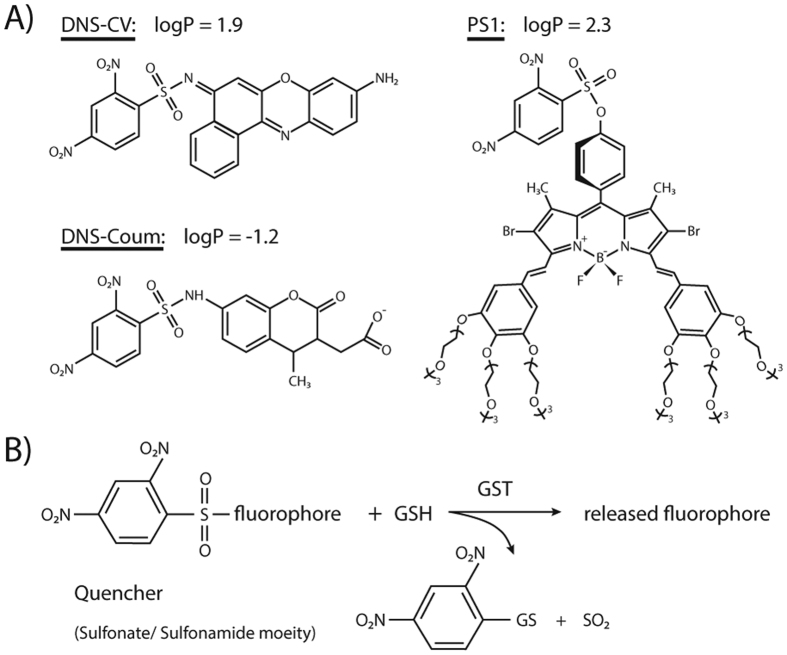
Chemcial structures and activation mechanism of the used GST substrates. (**A**) Chemical structures as well as theoretical logP values at pH 6.5. (**B**) All compounds are activated based on the sulfonamide/sulfonate cleavage activity of GSTs producing a GSH conjugate of the quencher moiety, SO_2_ and the released fluorophore.

When measuring the activity for soluble GSTs we first used a relatively hydrophilic substrate (DNs-Coum^9^, logP ≈ −1.2). As expected, the inclusion of detergent did not significantly alter the catalytic rate for a substrate that does not tend to partition into the detergent phase (“with 0.1% Triton X-100”: 261 ± 10 nmol/min mg; “without Triton X-100”: 208 ± 13 nmol/min mg (for GSTP1)). However, as the hydrophobicity of the substrate increases (DNs-CV^9^, logP ≈ 1.9) activity in the presence of detergent decreases by up to 100-fold (consistent with that predicted from the partition coefficient) (Fig. 1 and Table 1). As the detergent itself does not inhibit the enzyme activity, partitioning of the bulk of the hydrophobic substrate apparently prevents direct access to the soluble enzyme.

**Figure 2:**
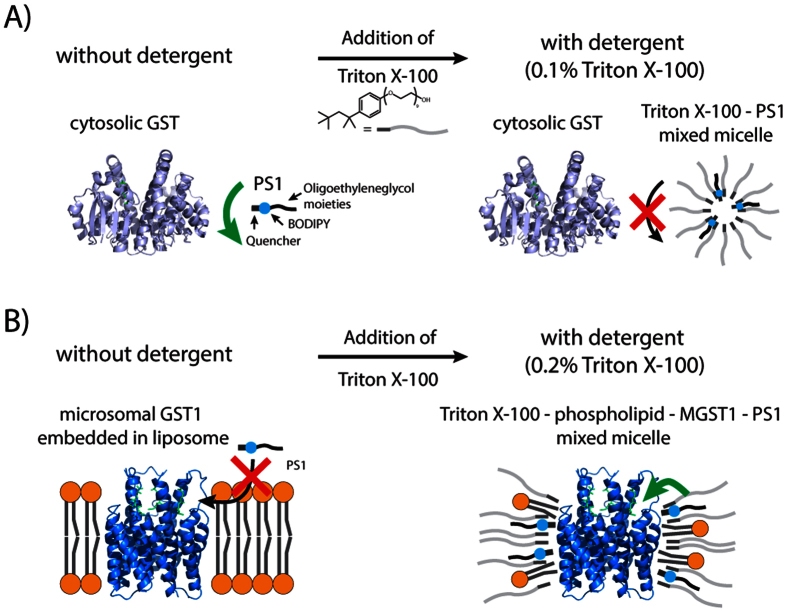
Schematic depiction of cytosolic and microsomal GSTs capacity to conjugate GSH to PS1 in the absence and presence of Triton X-100. (**A**) Left panel: Oligoethyleneglycol moieties of PS1 increase the water solubility of the fluorophore by preventing self-aggregation, thus enabling access to the active site of cytosolic GSTs in detergent free phosphate buffer. Right panel: By adding Triton X-100, PS1 forms mixed micelles with the detergent, effectively sequestering it from cytosolic GSTs. (**B**) Left panel: PS1 is not able to reach the active site of microsomal GSTs that are imbedded in lipid bilayers of liposomal preparations, consistent with its large size (MW = 1872 Da) and supported by the lack of fluorescence increase expected when PS1 transfers to hydrophobic media (Supporting Information). Right Panel: When Triton X-100 is added, mixed micelles comprised of Triton X-100, the microsomal GST, PS1 and lipids are formed. These conditions enable access of PS1 to the active site of the microsomal GST.

For the BODIPY-derived substrate PS1 this effect is most dramatic. The enzyme can access this substrate in the absence of detergent, but there is no activity detectable in the presence of detergent (Table 1)^10^. This property is consistent with the amphipathic nature of PS1 where oligooxyethylene groups, that are attached to increase water solubility, effectively shield the hydrophobic electrophilic site in the mixed micelle from enzyme access (Fig. 1 and illustrated in Fig. 2A; further discussed in the Appendix). Interestingly, the chemical background reaction towards GSH is not inhibited, but rather augmented 6-fold (Supplementary Table S2) showing that the PS1 electrophilic site is reactive and even more accessible to small molecules in the mixed micelle. We conclude that cytosolic enzymes, acting on hydrophobic substrates access the fraction of the substrate that resides in the aqueous phase. This conclusion is supported by several observations on the catalytic behavior of soluble enzymes in systems with two phases^11^,^12^,^13^,^14^. Notwithstanding these statements there are several soluble enzymes that act on membrane embedded substrates after binding to the membrane, in fact some members of the soluble enzymes (GSTs) have been described to exhibit this property (further discussed in the Appendix)^15^,^16^,^17^,^18^,^19^,^20^.

### Conversion of lipophilic substrates by membrane bound GSTs

In the case of integral membrane enzymes the active site can in principle point outside of the membrane, reside in the lipid headgroup/hydrocarbon interphase or be located exclusively in the hydrocarbon layer. For membrane enzymes acting broadly on hydrophobic substrates (like GSTs), however, a logical location would be the headgroup/hydrocarbon interphase region as the physicochemical properties of this region would favor hydrophobic interactions to the enzyme and thus efficient binding of hydrophobic substrates. For the membrane bound GST 1 (MGST1) and a closely related protein in the MAPEG superfamily (MPGES1) the crystal structures place the active sites precisely in this region^8^,^21^. Also, most substrates for MGST1 have hydrogen bonding capacity and, although largely hydrophobic, molecules with these properties tend to accumulate in this region^22^,^23^. To determine the principle access path to the active site of MGST1 (either directly via the cytosolic phase or via the hydrophobic inter-membrane phase) we devised experiments based on the unique properties of PS1.

PS1 has a bulky structure containing very hydrophilic oligoethyleneglycol moieties as well as a fairly high molecular weight that prevents it from being integrated into the ordered lipid bilayer of liposomes spontaneously (supported by the fact that PS1 does not develop fluorescence in the presence of liposomes as would be expected should it integrate; further discussed in the Appendix; Fig. 1A and illustrated in Fig. 2B, left panel)^10^. We used this property to ask whether MGST1, when incorporated into proteoliposomes^24^, could access PS1 in the aqueous phase where cytosolic GSTs do access PS1 (see above). This was clearly not the case (Table 2). To determine whether the enzyme could catalyze PS1 conversion in a hydrophobic environment, activity was measured in the presence of detergent. By adding Triton X-100, the ordered lipid structure of proteoliposomes is disturbed leading to the formation of mixed micelles that constitute MGST1, phospholipids and the detergent. Under these conditions PS1 was able to move into the detergent phase and to access the active side of proteoliposomal MGST1 (illustrated in Fig. 2B, right panel) with the enzyme effectively catalyzing the conjugation to GSH. The same behavior was observed with purified enzyme in the presence of detergent. In contrast to PS1, smaller hydrophobic substrates (e.g. CDNB) that readily partition into membranes do display activity with proteoliposomal MGST1 in the absence of added detergent (Table 2). Our results presented here outline that a potential substrate for MGST1 needs to access the hydrophobic environment and places the active site (access path for hydrophobic substrates) of the enzyme within the membrane, but does not specify where. Previous data using chloronitroaryl substrates with equal reactivity and varied hydrophobicity support that the active site of MGST1 is located in a hydrophilic phase within the membrane supporting the location in the headgroup/hydrocarbon interphase region^14^.

### PS1 as a substrate to assay GSTs *in vitro* and its intracellular activation for tumor treatment

There are 17 soluble and 3 membrane-bound GSTs in humans that display broad and overlapping substrate specificity^1^,^25^. The great catalytic versatility of GSTs is certainly important for protecting the organism, but makes it difficult to achieve analytical specificity for measuring individual enzymes or to target them individually as a means for anti-cancer therapy. PS1 displays a very high activity with MGST1 also compared to MGST2 and 3 (Table 2 and Supplementary Tables S1 and S2 in the Appendix). By including detergent in the assay MGST1 can thus be specifically quantified in cell extracts. Conversely, by omitting detergent only cytosolic GSTs will be assayed. PS1 was initially developed as a GSH cleavable photosensitizer to achieve high efficiency in photodynamic tumor cell treatment and does enter cells^10^. Our data showing that GSH dependent cleavage is an enzyme mediated process and characterizing substrate specificity of various GSTs (Table 1) (that are often overexpressed in tumors) suggest that targeting GSTA or GSTM overexpressing tumors should be more efficient^25^,^26^,^27^.

### Hydrophobic substrates and enzyme catalysis

Evolution of enzymes handling hydrophobic substances has resulted in complex pathways with membrane bound and soluble enzymes catalyzing various steps. Certainly chance has dictated some of what we see today. However, it appears that the early steps (involving the most hydrophobic substrates) are catalyzed by membrane bound enzymes (cytochrome P450s in the case of xenobiotic metabolism as well as cyclooxygenases and some lipoxygenases in eicosanoid metabolism)^28^,^29^,^30^,^31^,^32^,^33^,^34^. As to the various secondary metabolites, complementary systems with both membrane and soluble enzyme families have evolved. The rationale in xenobiotic metabolism is the need for efficient removal of toxic and reactive intermediates. The strategic location of an active site that faces the membrane (headgroup/hydrocarbon interphase) allows for a significant advantage and was actually demonstrated in a key experiment for MGST1 where, in whole cells, a reactive hydrophobic substrate was preferentially detoxified via the membrane pathway, although similar metabolic capacity was present in the soluble and membrane bound compartments^35^. In eicosanoid metabolism, originating from arachidonic acid release, we can speculate that the regulation and control of the competing reactions that produce different mediators and hence physiological outcome can be dictated by membrane location and also co-location of enzymes (known examples are COX2 and MPGES1)^36^.

Forneris and Mattevi review theoretical access paths of lipophilic substrates to membrane and soluble catalysts based on structures of relevant enzymes^5^. Here we provide an experimental approach to obtain data that can determine aqueous or membrane access paths. We show that the same substrate (PS1) can only be accessed from the enzymes native compartment. Although these results could be anticipated, rigorous experimental proof has so far been lacking. The basic principles of our approach could apply to other enzyme classes that have members in one or both phases and used to determine access paths or to validate the principle location of the active site. It might be feasible to attach a rather bulky and/or polyoxyethylene groups to a known substrate in order to prevent partitioning into the membrane to test whether the respective membrane bound enzyme has access to it. Certainly xenobiotic metabolizing enzymes (e.g. sulphotransferases/UDP-glucronysyl transferases) have a broad substrate specificity that should make the development of substrates with similar detergent dependent properties as PS1 feasible. To our knowledge, this strategy of preventing a substrate from spontaneous incorporation into phospholipid membranes and using detergent to prevent its interaction with soluble enzymes is presently the only way to solve the classical conundrum of demonstrating lipophilic substrate access paths to enzymes. Using an approach altering either substrate or lipid/detergent concentrations by necessity always results in parallel substrate concentration changes in both phases^14^ and is of no diagnostic value.

Additionally we discuss the advantage of placing an unspecific hydrophobic binding site in the membrane headgroup/hydrocarbon interphase where hydrophobic interactions can be utilized and many lipophilic compounds (containing some functionalities) do accumulate. Finally, the fluorogenic substrate PS1, that allowed us to perform these studies, can be used for specific determination of cytosolic vs. membrane bound GSTs.

## Materials and Methods

### Synthesis of fluorogenic compounds

The BODIPY-based sensitizer PS1 was synthesised as previously described by Turan *et al*. and solubilized in DMSO to a concentration of 250 mM^10^. Dilutions were made in 0.1 M phosphate buffer pH 6.5 with 0.1% Triton X-100 to keep the DMSO concentration below 1% in subsequent enzyme activity assays. The concentration of PS1 was determined spectrophotometrically by measuring the absorbance at 675 nm in PBS with 50% DMSO. 20 μM PS1 are hereby equal to an absorbance of 0.25 after baseline correction according to Turan *et al*.^10^.

### Determination of theoretical logP values

LogD values of all compounds were calculated using the “Physico-chemical property predictors” online software bundle provided by ChemAxon (https://www.chemaxon.com/products/calculator-plugins/property-predictors/). LogP was retained as logD at a pH of 6.5.

### Enzyme preparation

Human GSTA1 was heterologously expressed from the pET-21a (+) vector in *E. coli* BL-21 DE3 cells (Novagen, Madison, WI) and purified from bacterial lysate using a HiTrap SP cation-exchange column (Amersham Biosciences) as described previously^37^. Human GSTM1 was heterologously expressed from the pKK-D vector^38^ in *E. coli* XL1-Blue cells (Strategene, La Jolla, CA) and purified by affinity chromatography as described previously^39^,^40^. Human GSTP1 and GSTT1 were expressed and purified as described previously^41^,^42^. The high purity of the enzymes was confirmed by SDS/PAGE stained with Commassie Brilliant Blue R-250. MGST1 was purified from male Sprague Dawley rat livers as described previously^43^, with the exception that 0.2% Triton X-100 was used in the last purification step. MGST2 was expressed and purified as described previously^44^. MGST3 cloned into a pPICZA vector N-terminal hexa-histidine construct via homologous recombination before transforming into P. pastoris KM71H cells using the Pichia EasyComp Transformation kit (Invitrogen). The resulting Mut^S^ strain was cultured using buffered minimal glycerol/methanol media as described in the *Pichia* Expression Kit user manual (Invitrogen, Catalog no. K1710–01). Cells were harvested by centrifugation (3000 g, 6 min) and disrupted by combining with glass beads (0.5 mm) inside a Bead Beater (Biospec Products, Bartlesville USA) that was operated on ice in 7 × 1 minute cycles separated by 5 minute rests. The resulting slurry was filtered through nylon net filters (180 mm, Millipore) and centrifuged (1,500 g, 10 min). Membrane bound proteins in the supernatant were solubilized via the addition of Triton X-100 (1%, v/v) and sodium deoxycholate (0.5%, w/v) before adjusting the pH to 7.8 with 1 M NaOH dropwise and stirring for 1 h on ice. After centrifugation (10,000 g, 10 min) the supernatant was decanted and loaded onto a 5 ml HisTrap HP column (GE Healthcare) using a peristaltic pump. The column was then washed with 10 column volumes of Buffer A (25 mM Tris, 0.5 M NaCl, 10% glycerol, 0.03% DDM, 0.5 mM DTT, 1 mM GSH, 40 mM Imidazole, pH 7.8) before eluting with 3 column volumes of buffer A containing 300 mM of imidazole, before exchanging to assay buffer (0.1 M phosphate buffer pH 6.5 + 0.1% Triton). Protein concentration of MGST1, MGST2 and MGST3 was determined using the using Bradford method with bovine serum albumin as standard^45^. The concentration of active cytosolic GSTs was determined by measuring their activity with standard substrates and comparison with literature values^41^,^46^. For details on the activity assay see below.

### Preparation of MGST1 containing proteoliposomes

Proteoliposomes are composed of POPC lipids (1-Palmitoyl-2-Oleoyl-sn-Glycero-3-Phosphocholine, Avanti Polar Lipids, Alabaster, AL). 1 mg of these phospholipids was dried under a stream of N_2_. The residue was solubilized in 10 μl of 20% Na-cholate (Sigma-Aldrich, St. Louis, MO). The resulting suspension was sonicated in an ultrasonic bath (Elma Schmidbauer GmbH, Singen, DE) under N_2_. The sonication process consisted of 6 sonication periods of 30 sec each with 10 sec intervals for cooling. Thereafter, 90 μl of cooled buffer (10 mM potassium phosphate pH 7.0, 20% glycerol, 50 mM KCl, 0.1 mM EDTA (all ingredients are from Sigma-Aldrich, St. Louis, MO)) was added, followed by addition of 1.5 μg of MGST1. At last, 346 μl of cooled buffer (10 mM potassium phosphate pH 8.0, 0.2% Triton X-100, 20% glycerol, 0.1 mM EDTA, 1 mM GSH, 0.1 M KCl (all ingredients are from Sigma-Aldrich, St. Louis, MO)) was added. The resulting lipid protein mixture was transferred into an equilibrated dialysis tube and kept for dialysis for 96 h against buffer (10 mM potassium phosphate pH 7.0, 1 mM GSH, 20% glycerol, 50 mM KCl, 0.1 mM EDTA) (2 changes/24 h) and additional 96 h against buffer (10 mM potassium phosphate pH 7.0, 20% glycerol, 50 mM KCl, 0.1 mM EDTA) (2 changes/24 h). Proteoliposomes were harvested and stored at 4 °C under N_2_.

### Measurement of GST activity with standard substrates

The specific activity of GSTA1, GSTP1 and GSTM1 was measured in a 100 μl cuvette with a Cary 60 UV-visible spectrophotometer (Agilent Technologies, Santa Clara, USA) by following the change in absorbance at 340 nm using 1 mM GSH (Sigma-Aldrich, St. Louis, MO) and 1 mM CDNB (Merck, Darmstadt, Germany) as second substrate respectively. GSTT1 was assayed using 10 mM GSH and 0.5 mM EPNP (Sigma-Aldrich, St. Louis, MO) at 360 nm. The molar extinction coefficient used for CDNB conjugation was 9.6 mM^−1^ cm^−1 ^47 and for EPNP conjugation 0,5 mM^−1^cm^−1 ^41. Activity measurements of cytosolic GSTs were performed at 30 °C in 0.1 M potassium phosphate buffer pH 6.5. All microsomal GSTs were assayed at RT in 0.1 M potassium phosphate buffer pH 6.5 containing 0.1% Triton X-100 (required for enzyme solubility, Sigma-Aldrich, St. Louis, MO) using 5 mM GSH and 0.5 mM CDNB. CDNB activity of MGST1 in liposome preparations was assayed in 0.1 M potassium phosphate buffer pH 6.5 as well as in 0.1 M potassium phosphate buffer pH 6.5 with 0.2% Triton X-100 to mimic the conditions of the PS1 assay. Enzymatic activities were calculated after correction for the non-enzymatic reaction and were in general agreement with the values reported previously^42^,^43^,^44^,^46^. All measurements were taken in triplicate and slopes were fitted using the Cary WinUV software package (Agilent Technologies, Santa Clara, USA). These measurements were performed in order to validate the activity of the enzyme preparations used to characterise the fluorogenic substrate PS1 as well as to estimate the concentration of active enzyme.

MGST1 and MGST2 showed specific activities that were in good agreement with previously published values^43^,^44^. It shall be noted that the activity of MGST1 can be substantially increased by modifications of its cysteine-49 residue, including oxidative modifications or sulfhydryl reactive substances such as N-ethylmaleimide (NEM) that can activate the enzyme 15-30-fold^43^,^48^. A modification of Cys-49 may also affect the activity towards various substrates differently^49^. MGST3 was previously reported to have no CDNB activity^50^. However, we and others (unpublished results, Rinaldo-Matthis, A.), show that it can catalyse the conjugation with CDNB, albeit to a very low extent compared to MGST1 and MGST2 (Table 1). An explanation for the difference in results might be the enzyme amounts as Jakobsson *et al*. used MGST3 containing microsomes to measure CDNB activity in a Triton X-100 free buffer, whereas we performed the assay with purified, recombinant enzyme in the presents of 0.1% Triton. Additionally we also measured the activity of GSTA1, GSTM1 and GSTP1 towards CDNB as well as GSTT1 towards EPNP and compared our measurements with published results in order to estimate the amount of active enzyme in our preparations^41^,^46^.

### Measurement of GST activity with PS1

The GSH mediated cleavage of the quencher moiety of PS1 was measured with a Shimadzu RF-510LC fluorescence spectrophotometer (Analytical Instruments Division, Kyoto, Japan) using 660 nm excitation and 685 nm emission filters with a 10 nm bandwidth. Microsomal GSTs were measured in 0.1 M potassium phosphate buffer pH 6.5 containing 0.1% Triton X-100 by monitoring the release of the fluorophore. The cytosolic GSTs and liposome preparations however, were assayed in an endpoint format. Briefly, enzymes were incubated with 50 μM PS1 and 2 mM GSH in 0.1 M potassium phosphate buffer pH 6.5 at RT for 0, 20, 40 and 60 min respectively. 50 μl of the reaction mixture was mixed with 50 μl 0.1 M potassium phosphate buffer pH 6.5 containing 0.2% Triton X-100 in case of the cytosolic GSTs and 0.4% Trion X-100 for the liposome preparations. Fluorescence was subsequently recorded and the specific activity calculated after correction for the non-enzymatic reaction. To estimate the non-enzymatic reaction 10 mM GSO_3_^−^ was added to a second, otherwise identical, sample in order to completely inhibit the enzyme activity. Calibration curves were established by following the reaction of PS1 and GSH to completion in order to quantify the response.

## Additional Information

**How to cite this article**: Cebula, M. *et al*. Catalytic Conversion of Lipophilic Substrates by Phase constrained Enzymes in the Aqueous or in the Membrane Phase. *Sci. Rep.* **6**, 38316; doi: 10.1038/srep38316 (2016).

**Publisher's note:** Springer Nature remains neutral with regard to jurisdictional claims in published maps and institutional affiliations.

## Supplementary Material

Supplementary Information

## Figures and Tables

**Table 1:** Specific activities of cytosolic GSTs in the presence and absence of detergent.

Triton X-100	DNs-CV [nmol/min mg]		PS1 [nmol/min mg]	
	0%	0.1%	0%	0.1%
GSTA1	3100 ± 64	38 ± 1	59 ± 5	N.D.
GSTM1	1700 ± 110	52 ± 2	1100 ± 87	N.D.
GSTP1	89 ± 5	0.7 ± 0.1	0.4 ± 0.2	N.D.
GSTT1	8 ± 1	0.25 ± 0.03	1.3 ± 0.8	N.D.

Specific activities of cytosolic GSTs catalyzing the reaction of GSH and DNs-Coum, DNs-CV as well as PS1. Values are mean ± SEM; N = 3. N.D. Not detected.

**Table 2:** Specific activities of microsomal GSTs as well as MGST1 incorporated into liposomes.

	TritonX-100	CDNB [µmol/min mg]	PS1 [µmol/min mg]
MGST1 in liposomes	0%	6.7 ± 0.2	N.D.
	0.2%	7.8 ± 0.2	2.6 ± 0.1
MGST1	0.1%	7.9 ± 0.2	24 ± 0.9
MGST2	0.1%	17 ± 0.3	0.78 ± 0.03
MGST3	0.1%	0.06 ± 0.01	0.67 ± 0.01

Specific activities of the microsomal GSTs and MGST1 incorporated into liposomes in catalyzing the reaction of GSH and CDNB as well as PS1. Liposome embedded MGST1 was assayed in the absence and presence of detergent. Values are mean ± SEM; N = 3. N.D. Not detected.
