# Supplementary material for: Catalytic Conversion of Lipophilic Substrates by Phase constrained Enzymes in the Aqueous or in the Membrane Phase
Source: Sci Rep. 2016 Dec 5;6:38316. doi: 10.1038/srep38316 (PMC5137027; doi:10.1038/srep38316)
Supplement: Supplementary Information [file srep38316-s1.pdf]

Supplementary Information

Catalytic Conversion of Lipophilic Substrates by Phase constrained Enzymes in the Aqueous or in the Membrane Phase

Marcus Cebula, Ilke Simsek Turan, Birgitta Sjödin, Madhuranayaki Thulasingam, Joseph Brock, Volodymyr Chmyrov, Jerker Widengren, Hiroshi Abe, Bengt Mannervik, Jesper Z. Haeggström, Agnes Rinaldo-Matthis, Engin U. Akkaya, Ralf Morgenstern*

Contents

… 1. Fluorescence properties of PS1 in the presence and absence of Triton X-100

... 2. PS1-Triton X-100 mixed micelle formation and physical properties of PS1

... 3. PS1 as a novel fluorogenic substrate for microsomal GSTs

... 4. Cytosolic GSTs in the catalytic conversion of membrane embedded substrates

... 5. Additional Materials and Methods relevant to the Supplements

1. Fluorescence properties of PS1 in the presence and absence of Triton X-100

Recently, the photosensetizer "PS1" was developed having a 2,4-dinitrobenzenesulfonate group used as electron sink to quench the excited state of the BODIPY-based core chromophore. GSH mediated cleavage of the quencher moiety activates the compound leading to an increase in fluorescence and singlet oxygen production. [1] While studying whether activation of PS1 can be catalysed by specific GSTs we discovered that it has very specific detergent dependant properties that make it an ideal compound to evaluate the principles of second substrate access to cytosolic and microsomal GSTs.

PS1 was previously characterized in 50% DMSO/PBS, which is however, not compatible with enzymes due to the high DMSO concentration. GSTs in particular are typically assayed in 0.1 M potassium phosphate buffer pH 6.5 with or without the addition of 0.1% Triton X-100, depending on their intracellular location. Characterizing the absorption and emission spectra of pure and activated PS1 in these buffers, both, the degree of absorption as well as the shape of the spectrum were strongly dependent on the presence of the detergent (Supplementary Fig. S1). Specifically, the fluorescence was completely quenched in the absence of detergent whereas the addition of 0.1% Triton X-100 yielded a 6 times stronger fluorescence compared to 50% DMSO/PBS. The characteristics of PS1 thus entail low fluorescence in the aqueous phase and higher fluorescence in the hydrophobic environment of PS1-Triton mixed micelles. A contributing factor to the low fluorescence in aqueous media could be aggregation and self-quenching. However, PS1 is readily soluble in phosphate buffer and an alternate possibility rather suggests that the low quantum yield might be caused by a rotation of vinyl bonds due to interactions between the flexible oligoethyleneglycol moieties in PS1. Zhu et al. described this effect while studying the effects of styryl groups bearing oligoethyleneglycol moieties on the water solubility and fluorescent quantum yield of BODIPY dyes. Compound "H" in their analogue series strongly resembled PS1 with respect to structure, absorption spectrum as well as having a low fluorescence quantum yield in PBS. Zhu et al. suggested that a strong absorption peak at 383 nm that is attributed to the phenyl units at the 3,5-position, a shoulder peak at 605 nm and a weak BODIPY core absorption at 507 nm – which all are also features represented in the absorption spectrum of PS1 (Supplementary Fig. S1) - support the notion of interacting oligoethyleneglycol moieties being the reason for the low quantum yield. [2] Furthermore, the interactions between the oligoethyleneglycol moieties are likely disrupted to some extent by DMSO and moreover by Triton X-100 when forming mixed PS1-Triton X-100 micelles, thus increasing the quantum yield and enabling the detection of fluorescence emission. This is also supported by the relative increase in absorbance at 660 nm when DMSO or Triton X-100 is present. A similar pattern was also described by Zhu *et al.* when studying the absorption and fluorescence quantum yield by compound H in Dichloromethane vs. PBS. [2]

2. PS1-Triton X-100 mixed micelle formation and physical properties of PS1

PS1 has an amphiphilic structure as it contains hydrophobic (BODIPY core and sulphonamide moiety) and hydrophilic oligoethyleneglycol moieties. These features are likely responsible for an efficient incorporation into Triton X-100 micelles, where PS1 is hidden from the active site of cytosolic enzymes (Fig. 2A and Table 1). We further confirmed the incorporation of PS1 (MW: 1872 Da) into Triton X-100 micelles using gel filtration col-

umns with an exclusion size of 6000 Da. 200 μM PS1 co-localised completely with Triton X-100 micelles as seen by the elution profile and the full recovery of PS1 in the exclusion volume (Supplementary Fig. S2A). This result suggests that there is no free PS1 in the presence of Triton X-100, which is well in line with the observation that cytosolic GSTs have no access to PS1 under this condition (Table 1).

In the absence of Triton X-100 we observed that PS1 is not a substrate for MGST1 embedded in liposomes (Fig. 2B and Table 2). The reason is that PS1, due to its large size (MW: 1872 Da) and bulky hydrophilic and hydrophobic sites, does not enter the lipid bilayer and therefore cannot gain access to the active site of the enzyme, which is further indicated by the lack of PS1 fluorescence in this setup.

We did not perform a detailed analysis of the physical properties of PS1 in the absence of detergent as the incorporation of PS1 into Triton X-100 micelles and the effect on the catalytic activity of cytosolic GSTs is very clear cut. Nonetheless, based on its amphiphilic nature PS1 might form micelles or aggregates. Such structures would prevent access of the sulphonamide to the active side of cytosolic enzymes thus affecting their catalytic activity. However, both, the hydrophobic and hydrophilic sites, are very bulky indicating that a significant contribution only occurs at higher concentrations. To estimate whether PS1 micelles or aggregates were formed at the concentrations used, we analysed whether PS1 also appears in the exclusion volume of the desalting columns (exclusion size of 6000 Da) in the absence of Triton X-100. At a concentration of 50 μM PS1 in the absence of Triton X-100 we noted that PS1 did not elute with the exclusion volume and also that it did not enter the resin. Instead it remained at the top of the column even after extensive washing with Triton X-100 free buffer - potentially due to interactions of its hydrophobic groups with the resin. However, when 0.1% Triton X-100 containing buffer was added to the column PS1 could be completely recovered, suggesting that PS1-Triton X-100 mixed micelles were formed and that the interactions of PS1 with the resin were effectively abrogated (Supplementary Fig. S2B). Interestingly, when loading 200 μM PS1 in the absence of Triton X-100 to the column approximately 43% of PS1 could be eluted in the exclusion volume, demonstrating that PS1 forms higher molecular weight structures at a concentration of approximately 100 μM and above. Analysing a second sample of 200 μM PS1 on the very same column, without having recovered the remaining PS1 with Triton X-100, we detected a similar elution profile with a recovery of 63% PS1 in the exclusion volume. The slight increase in PS1 recovery is likely a consequence of saturation of the interactions of free PS1 with the resin on top of the column after loading the first PS1 sample, which thus shifted the equilibrium of free PS1 and higher molecular weight forms of PS1 to the latter (Supplementary Fig. 2C). These data do not support that PS1 forms micelles or aggregates at the lower concentrations used in our assays. It appears that at most, higher molecular aggregates comprise a minor fraction and consequently would not impede the catalytic activity of cytosolic GSTs.

3. PS1 as a fluorogenic substrate for microsomal GSTs

Despite extensive research, sensitive detection and selective targeting of individual GSTs is still difficult to achieve - partly due to their broad substrate specificity, but also in respect to the vast number of individual GSTs within the cell, underlining the need for an improved understanding of the substrate selectivity determinants, access and recognition. For PS1, the exclusive specificity for microsomal GSTs in the presence of Triton X-100 is

unique among all known fluorogenic substrates, enabling to distinguish between cytosolic and microsomal GST activity in complex biologic samples. To further assess whether PS1 is a suitable candidate to assay GST activity we performed steady-state kinetic analyses on MGST 1-3 with respect to PS1 as well as GSH (Supplementary Table 1). Of all three enzymes, MGST1 had the lowest $K_{m, app}$ (9.3 μM) and highest k_{cat} (25 s^{-1}) values, resulting in an efficiency of 10^6 $M^{-1}s^{-1}$, which may be compared to the standard substrate CDNB (10^5 $M^{-1}s^{-1}$ for MGST1) [3-5] or to the fluorogenic substrate DNs-CV (10^5 $M^{-1}s^{-1}$ for MGST1) [6] and DNs-Coum (10^3 $M^{-1}s^{-1}$ for MGST1) [6] thus showing that it is one of most efficient substrates for microsomal GST1 characterised so far. This is also reflected by the rate enhancements that are in the order of 10^8 for MGST1 and 10^6 for the MGST2 and MGST3 respectively (Supplementary Table S2). By altering the hydrophobic properties of PS1, [6,7] fine-tuning its reactivity [3,6] and optimising the gain of fluorescence upon activation, suitable PS1 based compounds can potentially be developed as MGST substrates and membrane targeted photosensitizers. As expected, PS1 being a sulfonic acid ester, the background rate (Supplementary Table S2) is in general higher than for the earlier characterized sulphonamide substrates. [6]

We wish to emphasize that the nominal substrate concentration was used to calculate the apparent K_m ($K_{m, app}$) and apparent k_{cat}/K_m ($k_{cat}/K_{m, app}$) values and that partitioning of the substrate was not taken into account. This is typically done as it makes no assumption of the effective substrate concentration in each phase (due to solubility and partitioning) and no assumption about the enzyme active site location. It is therefore logical that efficient substrates for MGST1 are those that are very hydrophobic such as PS1 or DNs-CV whereas the less hydrophobic substrates are less efficient (e.g. DNs-Coum). [6]

In addition, the properties of PS1, particularly the lack of fluorescence in a detergent free environment can be an advantage for its use in photodynamic therapy. In the non-fluorescent state the molecule does not lose its excitation energy, which increases the life-time of an excited singlet or triplet state thus enhancing the probability for photochemistry with oxygen and singlet oxygen production. [8] Additionally, targeting cancers that overexpress GSTA or GSTM may enable specificity in photodynamic therapy.

4. Cytosolic GSTs in the catalytic conversion of membrane embedded substrates

Certain GSTs of the alpha class are known to directly reduce phospholipid hydroperoxides (PL-OOH), which are still embedded in the membrane in order to mitigate lipid peroxidation. Being cytosolic enzymes, it was first suggested that they rely on the phospholipase A_2-mediated release of fatty acid hydroperoxides (FA-OOH) from membrane embedded PL-OOH, but it was later shown not to be a requirement. [9-12] The underlying processes have not yet been delineated, but it seems rather unlikely that the enzyme can access the hydrophobic interior of the membrane. A potential mechanism could be that the oxidized group of the PL-OOH gains access to the active site of the cytosolic GST at the phospholipid-headgroup/ hydrocarbon border, thus circumventing the thermodynamic problem that the enzyme encounters within the lipid bilayer. A recent study showed that oxidized groups in lipid tails promoted conformational changes where the oxidized tails bend toward the water phase with the oxygen atoms forming hydrogen bonds with water and the polar lipid headgroup. [13] Incidentally, cytosolic GSTs of the alpha class as well as mu and pi, have been found to partly attach to the cytoplasmic periphery of membranes. [14-

[17] This localization might not only enable an efficient reduction of PL-OOHs in membranes without the need of their release into the aquatic phase, but furthermore also of other hydrophobic electrophiles that are expected to accumulate at the phospholipid-headgroup/fatty-acid-hydrocarbon chain intersection.

5. Additional Materials and Methods relevant to the Supplementary Information

Spectroscopic characterization of PS1. Absorption spectra were measured in a 100 µl cuvette with a Cary 60 UV-visible spectrophotometer using 25 µM PS1 and 2 mM GSH respectively. The absorbance of the buffer was subtracted as baseline. Excitation and emission spectra were obtained using an EnSpire 2300 Multilabel Reader (Perkin Elmer). All measurements were performed in a 96-well optical bottom plate (Fisher Scientific, #265301) with 25 µM PS1 and 2 mM GSH in a 200 µl volume. Measurements were taken at room temperature in 0.1 M potassium phosphate buffer pH 6.5 containing either no additional Triton X-100 or with 0.1% Triton X-100 as well as in PBS containing 50% DMSO.

Analysis of PS1 and mixed PS1-Triton X-100 micelle formation. The elution profile of PS1 (MW: 1872 Da) was analysed using pre-packed Econo-Pac 10DG desalting columns with an exclusion size of 6000 dalton (Bio-Rad) according to the manufacturer's guidelines. Briefly, the column was equilibrated using 25 ml of buffer. After the buffer drained to the top frit 0.1 ml of 50 µM PS1 was added and allowed to enter the column before an additional 2.5 ml buffer was added with the effluent being discarded. Next, 0.1 ml of buffer was added stepwise and the corresponding 0.1 ml fractions of effluent collected for further analysis. The experiments were performed using 0.1 M potassium phosphate buffer pH 6.5 either with or without 0.1% Triton X-100. The PS1 content was determined via absorbance at 675 nm using a Cary 60 UV-visible spectrophotometer. An aliquot of the sample before adding to the column was analysed as control. Before absorbance measurements, 0.1 ml of either 0.1% or 0.2% Triton X-100 containing 0.1 M potassium phosphate buffer pH 6.5 were added to the corresponding 0.1 ml effluent fractions to have a final concentration of 0.1% Triton X-100 in all samples as well as a comparable volume.

The elution profile of Triton X-100 was determined by analysing 0.1 ml of 0.1% Triton X-100 (0.1 M potassium phosphate buffer pH 6.5) in the background of 0.02% Triton X-100 containing 0.1 M potassium phosphate buffer pH 6.5 that was used to equilibrate the column and to elute the sample. Triton X-100 content was determined via absorbance at 275 nm using a Cary 60 UV-visible spectrophotometer.

Determination of Steady-State Kinetic Constants. The kinetic parameters k_{cat}, apparent K_m ($K_{m,\ app}$) and apparent k_{cat}/K_m ($k_{cat}/K_{m,\ app}$) were determined for the microsomal GSTs (MGST1, MGST2 and MGST3) by fitting the Michaelis–Menten equation to the data by nonlinear regression analysis using GraphPad Prism 5 (GraphPad Software, Inc., San Diego, CA). Saturation could not be reached in all cases, wherefore we also determined $k_{cat}/K_{m,\ app}$ by fitting the equation $v = (k_{cat}/K_{m,\ app})[E][S]$ to the low substrate concentration data points ($[S] < K_{m,\ app}$). For the calculation of the turnover we took into account that microsomal GSTs display one-third of the sites reactivity and calculated k_{cat} per trimer (MGST1: 52.7 kDa; MGST2: 49.9 kDa; MGST3: 49.5 kDa). [18] All micro-

somal GSTs were assayed at either a constant GSH concentration of 2 mM with varying PS1 concentrations (1 - 50 μM) or at a constant PS1 concentration of 25 μM with varying GSH concentrations (0.05 - 4 mM) respectively. The nominal substrate concentration was used to calculate the apparent K_m ($K_{m, app}$) and apparent k_{cat}/K_m ($k_{cat}/K_{m, app}$) values. The enzyme concentration within the Triton X-100 micelles and the effective substrate concentration in these micelles was not taken into account.

REFERENCES

1 Turan, I. S., Cakmak, F. P., Yildirim, D. C., Cetin-Atalay, R. & Akkaya, E. U. Near-IR absorbing BODIPY derivatives as glutathione-activated photosensitizers for selective photodynamic action. *Chemistry* **20**, 16088-16092, doi:10.1002/chem.201405450 (2014).

2 Zhu, S. L. *et al.* Highly Water-Soluble Neutral BODIPY Dyes with Controllable Fluorescence Quantum Yields. *Organic Letters* **13**, 438-441, doi:10.1021/ol102758z (2011).

3 Morgenstern, R., Lundqvist, G., Hancock, V. & DePierre, J. W. Studies on the activity and activation of rat liver microsomal glutathione transferase, in particular with a substrate analogue series. *J Biol Chem* **263**, 6671-6675 (1988).

4 Andersson, C. *et al.* Kinetic studies on rat liver microsomal glutathione transferase: consequences of activation. *Biochim Biophys Acta* **1247**, 277-283 (1995).

5 Ahmad, S., Dalwai, A. & Al-Nakib, W. Frequency of enterovirus detection in blood samples of neonates admitted to hospital with sepsis-like illness in Kuwait. *J Med Virol* **85**, 1280-1285, doi:10.1002/jmv.23604 (2013).

6 Zhang, J. *et al.* Synthesis and characterization of a series of highly fluorogenic substrates for glutathione transferases, a general strategy. *J Am Chem Soc* **133**, 14109-14119, doi:10.1021/ja205500y (2011).

7 Morgenstern, R. A simple alternate substrate test can help determine the aqueous or bilayer location of binding sites for hydrophobic ligands/substrates on membrane proteins. *Chem Res Toxicol* **11**, 703-707, doi:10.1021/tx980013e (1998).

8 Foote, C. S. Mechanisms of photosensitized oxidation. There are several different types of photosensitized oxidation which may be important in biological systems. *Science* **162**, 963-970 (1968).

9 Tan, K. H., Meyer, D. J., Belin, J. & Ketterer, B. Inhibition of Microsomal Lipid-Peroxidation by Glutathione and Glutathione Transferase-B and Transferase-Aa - Role of Endogenous Phospholipase-A2. *Biochemical Journal* **220**, 243-252 (1984).

10 Yang, Y. *et al.* Role of glutathione S-transferases in protection against lipid peroxidation - Overexpression of hgsta2-2 in k562 cells protects against hydrogen peroxide-induced apoptosis and inhibits JNK and caspase 3 activation. *Journal of Biological Chemistry* **276**, 19220-19230, doi:DOI 10.1074/jbc.M100551200 (2001).

11 Yang, Y. S. *et al.* Protection of HLE B-3 cells against hydrogen peroxide- and naphthalene-induced lipid peroxidation and apoptosis by transfection with hGSTA1 and hGSTA2. *Invest Ophth Vis Sci* **43**, 434-445 (2002).

12 Yang, Y. S., Sharma, R., Zimniak, P. & Awasthi, Y. C. Role of alpha class glutathione S-transferases as antioxidant enzymes in rodent tissues. *Toxicol Appl Pharm* **182**, 105-115, doi:10.1006/taap.2002.9450 (2002).

13 Wong-Ekkabut, J. *et al.* Effect of lipid peroxidation on the properties of lipid bilayers: A molecular dynamics study. *Biophys J* **93**, 4225-4236, doi:10.1529/biophysj.107.112565 (2007).

14 Singh, S. P. *et al.* Membrane association of glutathione S-transferase mGSTA4-4, an enzyme that metabolizes lipid peroxidation products. *Journal of Biological Chemistry* **277**, 4232-4239, doi:10.1074/jbc.M109678200 (2002).

15 Robin, M. A., Prabu, S. K., Raza, H., Anandatheerthavarada, H. K. & Avadhani, N. G. Phosphorylation enhances mitochondrial targeting of GSTA4-4 through increased affinity for binding to cytoplasmic Hsp70. *Journal of Biological Chemistry* **278**, 18960-18970, doi:10.1074/jbc.M301807200 (2003).

16 Hemachand, T., Gopalakrishnan, B., Salunke, D. M., Totey, S. M. & Shaha, C. Sperm plasma-membrane-associated glutathione S-transferases as gamete recognition molecules. *Journal of Cell Science* **115**, 2053-2065 (2002).

17 Morgenstern, R., Guthenberg, C., Mannervik, B. & DePierre, J. W. The amount and nature of glutathione transferases in rat liver microsomes determined by immunochemical methods. *FEBS Lett* **160**, 264-268 (1983).

18 Ricci, G. *et al.* Colorimetric and fluorometric assays of glutathione transferase based on 7-chloro-4-nitrobenzo-2-oxa-1,3-diazole. *Anal Biochem* **218**, 463-465 (1994).

Supplementary Figure Legends

Supplementary Figure S1. Absorption and emission spectra of PS1. A) Absorption and B) emission spectra of 25 µM PS1 and the product after reaction with 2 mM GSH for up to 90 min in PBS + 50% DMSO as well as 0.1 M phosphate buffer pH 6.5 with and without 0.1% Triton X-100. Excitation wavelength was 440 nm.

Supplementary Figure S2. Elution profile of PS1 in the absence and presence of 0.1% Triton X-100. A) Elution profile of 0.1 ml of 200 µM PS1 in 0.1% Triton X-100 containing 0.1 M phosphate buffer pH 6.5 (dark grey bars) as well as of 0.1 ml 0.1% Triton in the background of 0.02% Triton containing buffer to study the elution profile of Triton (light grey bars) in order to determine if PS1 co-migrates in mixed micelles. **B)** 0.1 ml of 50 µM PS1 in 0.1 M phosphate buffer pH 6.5 was loaded to an Econo-Pac 10DG desalting columns with an exclusion size of 6000 dalton. Elution was performed first in the absence of Triton X-100, followed by a second elution using 0.1 % Triton X-100 containing buffer. **C)** Elution profile of 0.1 ml 200 µM PS1 in the absence of Triton X-100. After a first elution 0.1 ml of 200 µM PS1 was additionally loaded and a second elution was performed. **A-C)** The effluent was collected in 0.1 ml fractions with the relevant fractions numbered to compare between experiments. PS1 content was determined via absorbance at 675 nm and Triton X-100 at 275 nm. Values are displayed relative to the total absorbance of the sample loaded and the integral was calculated to determine the fraction of PS1 that was eluted. Values are mean ± SEM (N = 3). **C)** was performed as a single experiment.

Supplementary Figure S1

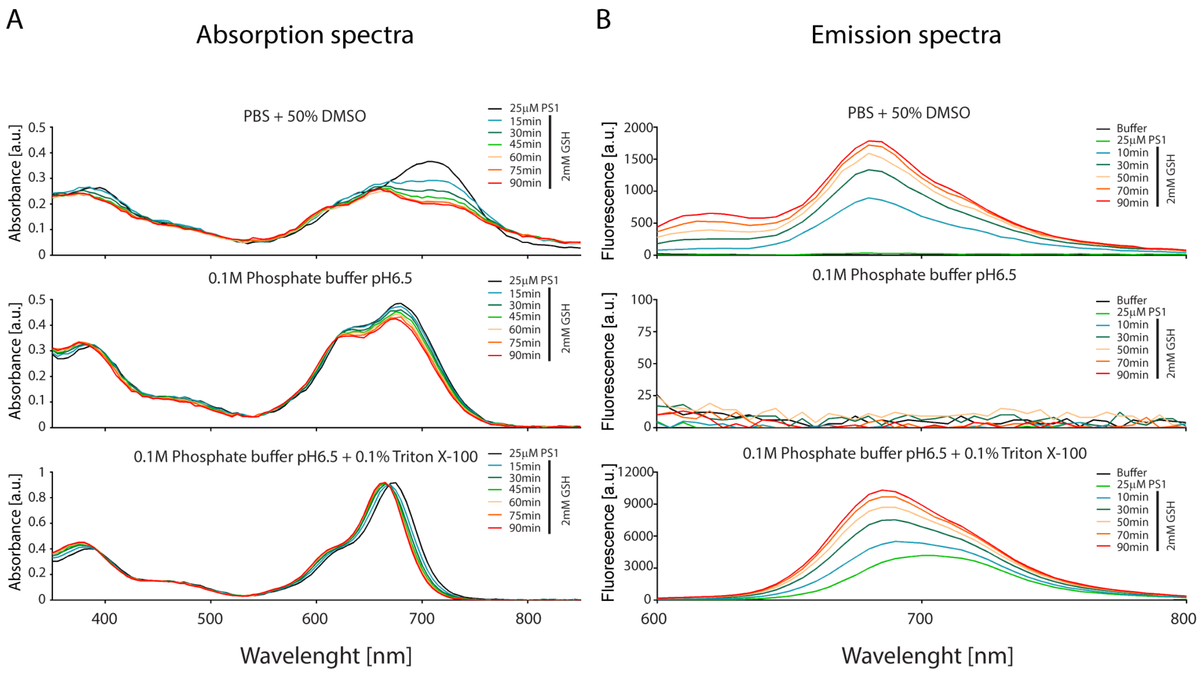

Supplementary Figure S2

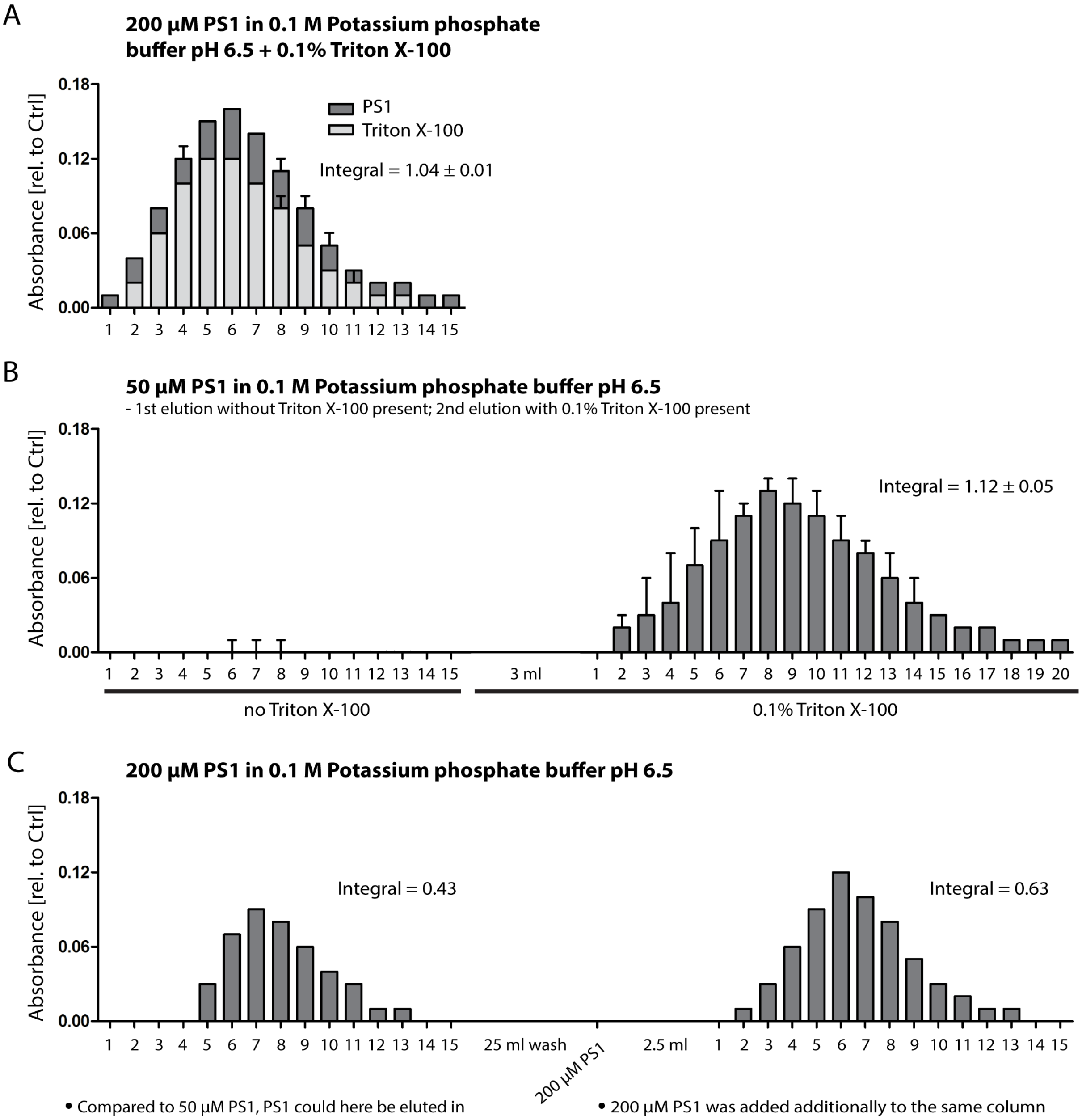

- Compared to 50 µM PS1, PS1 could here be eluted in the absense of Triton X-100. The fraction of 43% PS1 in the efflux potentially relates to higher molecular weight forms of PS1 such as micelles or aggregates.
- 200 µM PS1 was added additionally to the same column after washing with 25 ml of Triton X-100 free buffer. Without having cleaned the column with 0.1% Triton X-100 part of the PS1 binding capacity was already saturated leading to a higher yield of PS1 in the absense of Triton X-100.

Supplementary Table S1. Steady-State kinetic parameters of microsomal GSTs with PS1 and GSH.

		k_{cat} [s^{-1}]	$K_{m, app}$ [µM]	$k_{cat}/K_{m, app}$ [$M^{-1}s^{-1}$]	$k_{cat}/K_{m, app}$ [$M^{-1}s^{-1}$] (via low substrate conc.)
**MGST1**	PS1	24.9 ± 0.9	9 ± 1	(2.7 ± 0.3) x 10^6	(2.6 ± 0.3) x 10^6
	GSH	21.3 ± 0.8	1230 ± 120	(1.7 ± 0.2) x 10^4	(1.7 ± 0.1) x 10^4
**MGST2**	PS1	0.65 ± 0.02	22 ± 2	(2.9 ± 0.3) x 10^4	(2.0 ± 0.1) x 10^4
	GSH	0.43 ± 0.01	430 ± 30	(9.9 ± 0.7) x 10^2	(6.2 ± 0.5) x 10^2
**MGST3**	PS1	0.99 ± 0.05	15 ± 2	(6.8 ± 0.8) x 10^4	(4.9 ± 0.1) x 10^4
	GSH	0.55 ± 0.01	220 ± 2	(2.5 ± 0.2) x 10^3	(2.0 ± 0.1) x 10^3

MGST1, MGST2 and MGST3 were assayed at either a constant PS1 concentration of 25 µM with varying GSH concentrations (0.05 - 4 mM) or at a constant GSH concentration of 2 mM with varying PS1 concentrations (1 - 50 µM) in 0.1 M potassium phosphate buffer pH 6.5 containing 0.1 % Triton X-100. The final DMSO concentration was < 0.1 %. At least six different concentrations were measured in triplicates per data set. Data sets were plotted in Graphpad Prism 5, fitted with the Michaelis-Menten equation by nonlinear regression and the kinetic parameters k_{cat}, $K_{m, app}$ and k_{cat}/K_m, app as well as their standard errors were obtained. Saturation kinetics were difficult to obtain due to the hydrophobic nature of PS1, which was only used up to a concentration of 50 µM. In order to validate our parameters we also obtained the $k_{cat}/K_{m, app}$ values by using the Michaelis-Menten relationship for low substrate concentrations: for [S] < K_m applies v/[E][S] = k_{cat}/K_m. $k_{cat}/K_{m, app}$ values and their respective standard errors were obtained by fitting the respective data points with a linear regression in Graphpad Prism 5.

Supplementary Table S2. Second order rate constants for the non-enzymatic reaction and rate enhancements by microsomal GSTs.

	k_{noncat} [M^{-1} s^{-1}]
**with 0.1% Triton X-100**	$(3.0 \pm 0.2) \times 10^{-2}$
**without 0.1% Triton X-100**	$(5.3 \pm 0.9) \times 10^{-3}$
**Rate enhancements by microsomal GSTs**	
**MGST1**	$(8.8 \pm 1.1) \times 10^{7}$
**MGST2**	$(9.6 \pm 1.0) \times 10^{5}$
**MGST3**	$(2.2 \pm 0.3) \times 10^{6}$

The second-order rate constant for the non-enzymatic reaction (k_{noncat}) was measured using 2 mM GSH and 50 μM PS1 in 0.1 M potassium phosphate buffer pH 6.5 with and without 0.1% Triton X-100. Values are mean ± SEM from two independent experiments, measured in duplicates (N = 4). Rate enhancements are based on the $k_{cat}/K_{m,\ app}$ values with respect to PS1 variation (see Table 2) and the non-enzymatic reaction in 0.1 M potassium phosphate buffer pH 6.5 with 0.1% Triton X-100.
